# Supplementary material for: Skeletons in the permafrost: Exploring climate-driven heritage loss and occupational health at the early modern whaling burial site of Likneset, Svalbard
Source: PLoS One. 2026 May 20;21(5):e0347033. doi: 10.1371/journal.pone.0347033 (PMC13189349; doi:10.1371/journal.pone.0347033)
Supplement: S1 Table — Word table listing all analyzed individuals from the Likneset burial site, including excavation phase, year, grave number, specimen or museum number, field area, excavation method, and summary information on osteological analysis and curation history. (DOCX) [file pone.0347033.s003.docx]

| Excavation phase (I, II, III) | Year | Grave no. | Individual ID / Museum no. | Field area (A/B) | Excavation method | Osteological analysis |
| --- | --- | --- | --- | --- | --- | --- |
| 1985-1990 | | | | | | |
| I | **1985** | **214** | **Ts.8940** | A | In situ | Thirteen of the fourteen skeletons (excluding Grave 211) were originally examined by Berit Sellevold in connection with the initial excavations (Sellevold 1993, 2000). New osteological analyses of these thirteen skeletons were conducted in 2023 by Elin T. Brødholt as part of the present project (Loktu & Brødholt 2024). The complete osteological report is available in Norwegian and can be downloaded here: https://www.niku.no/wp-content/uploads/2025/01/NIKU-Rapport-427-Skjeletter-i-skapet-Likneset.pdf |
| I | **1985** | **215** | **Ts.8941** | A | In situ |  |
| I | **1985** | **217** | **Ts.8944** | A | In situ |  |
| I | **1985** | **220** | **Ts.8945** | A | In situ |  |
| I | **1985** | **216 A** | **Ts.8942** | A | Partly block lift |  |
| I | **1985** | **216 B** | **Ts.8943** | A | In situ |  |
| I | **1986** | **219** | **Ts.8946** | A | Block lift |  |
| I | **1986** | **222** | **Ts.8947** | A | Block lift |  |
| I | **1989** | **212** | **Ts.8948** | A | Partly block lift |  |
| I | **1989** | **213** | **Ts.8949** | A | Partly block lift |  |
| I | **1989** | **221** | **Ts.8950** | A | In situ |  |
| I | **1989** | **223** | **Ts.8951** | A | In situ |  |
| I | **1990** | **211** | **Ts. 8972** | A | In situ |  |
| I | **1990** | **218** | **Ts. 8973** | A | Block lift |  |
| 2016 | | | | | | |
| II | **2016** | **201** | **SVB 07553** | A | Block lift | Osteological analyses were carried out in connection with the archaeological excavation project at Likneset in 2016 (see Loktu & Vivås 2022). The analyses were conducted by Elin T. Brødholt. |
| II | **2016** | **200** | **SVB 07554** | A | Block lift |  |
| II | **2016** | **202** | **SVB 07552** | A | Block lift |  |
| 2019 | | | | | | |
| III | **2019** | **66** | **SVB 9927** | B | Block lift | Osteological analyses were carried out in connection with the archaeological excavation project at Likneset in 2019 (see Loktu & Bjerkestrand, in prep.). Due to delays caused by the COVID-19 pandemic, the analyses were conducted in January 2023 by Elin T. Brødholt. |
| III | **2019** | **1** | **SVB 9929** | B | Block lift |  |
| III | **2019** | **78** | **SVB 9928** | B | Block lift |  |

**Table. Repository and specimen numbers, excavation history, and osteological analysis overview for all individuals from the Likneset burial site (ID 93705)**

Material excavated in 2016 [73] and 2019 [74] is curated under museum accession numbers SVB 7552–7554 and SVB 9927–9929, respectively. Phase I material remains curated under the original Ts. accession numbers (8940–8951, 8972, 8973), pending full re-accessioning from the Arctic University Museum of Norway (UiT). Field documentation, excavation reports, conservation records, and photographic archives are held at the Svalbard Museum and by The Governor of Svalbard.
