# Supplementary material for: Skeletons in the permafrost: Exploring climate-driven heritage loss and occupational health at the early modern whaling burial site of Likneset, Svalbard
Source: PLoS One. 2026 May 20;21(5):e0347033. doi: 10.1371/journal.pone.0347033 (PMC13189349; doi:10.1371/journal.pone.0347033)
Supplement: Attachment — Submitted filename: Respons to Reviewers - PLOSone.docx [file pone.0347033.s005.docx]

**Response to Reviewers - [PONE-D-25-37512]**

Dear Academic Editor and Reviewers,

We thank the Academic Editor and both reviewers for their thorough and constructive evaluations of our manuscript *Skeletons in the Permafrost: Exploring Climate-Driven Heritage Loss and Occupational Health at an Early Modern Whaling Burial Site at Likneset, Svalbard.* We appreciate the recognition that the Likneset burial site represents a unique and valuable case study and that the topic is timely and relevant.

In response to the major revision decision, we have undertaken a comprehensive reworking of the manuscript relative to the original submission to address all points raised. The text has been restructured to ensure a clear separation between Results and Discussion, and the Methods section has been substantially expanded to improve methodological transparency and analytical clarity. The analytical framework is now explicitly defined and supported by a semi-quantitative preservation scoring system presented in a dedicated table.

The research questions and corresponding results have been clarified and consistently integrated throughout the manuscript. To strengthen data accessibility and reproducibility, we have added S1 Appendix A (preservation conditions and scores) and S2 Appendix B (individual-level osteological data), together with a comprehensive overview of the analysed material, including grave identifiers and museum catalogue numbers in S3 Table. We have also revised and streamlined the text by expanding previously underdeveloped arguments directly relevant to the analysis, while removing sections that were not strictly necessary for addressing the stated research aims. All figures have been revised accordingly.

Given the extent of the revisions, including structural reorganisation and substantial rewriting of multiple sections, tracked changes in the revised manuscript primarily reflect major structural and content-related modifications rather than line-by-line edits.

Below, we respond point by point to the Academic Editor’s summary comments and to each reviewer’s detailed remarks.

Thank you for considering our submission.

**Sincerely,**
Lise Loktu
Corresponding author
Norwegian Institute for Cultural Heritage Research (NIKU)
E-mail: lise.loktu@niku.no

**Response to Reviewers – [PONE-D-25-37512]**

| **Reviewer / Editor comment** | **Response** | **Location in revised manuscript** |
| --- | --- | --- |
| **Academic Editor:** Manuscript reads largely as a descriptive site report with limited integration between aims, methods, and results. | The manuscript has been substantially revised to strengthen analytical integration. Aims, methods, and results are now explicitly aligned, with Results structured according to the two research questions (RQ1 and RQ2), and Discussion synthesising findings without introducing new data. | Introduction; Methods (Analytical framework); Results (section structure); Discussion |
| **Academic Editor:** Limited integration between research aims, methods, and results. | This integration has been clarified throughout. The analytical framework is now explicitly designed to address the stated research questions and enable comparison across excavation phases and burial microenvironments. | Introduction; Methods; Results |
| **Academic Editor:** Need clearer methodological detail regarding sampling strategy, data comparability across excavation phases, and taphonomic recording. | We now explicitly state that all 19 individuals included in the osteological analyses were reassessed by the same analyst using identical protocols, including material excavated in the 1980s. A detailed semi-quantitative preservation and taphonomic scoring system is presented, with variables defined in Table 1 and individual-level data provided in the Supplementary Data. | Methods: Osteological methods; Analytical framework and preservation assessment; Table 1; Supplementary Data (Appendices A–B) |
| **Academic Editor:** Need a more explicit, evidence-based link between observed degradation and climate-related processes. | The manuscript now clarifies that the study documents empirical preservation change rather than direct climatic causation. Comparison between Phase I (1985–1990) and Phase II (2016) burials is explicitly restricted to graves located in close spatial proximity within the same erosion-exposed sector (Field area A), documenting continued in situ degradation under broadly comparable microenvironmental conditions. This point is now clearly stated in the Abstract, Results, and Conclusion. | Abstract; Results (RQ1); Conclusion |
| **Academic Editor:** Need more quantitative data. | Quantitative and semi-quantitative data are now presented through tables and supplementary appendices, including prevalence values and preservation scores. | Results; Tables; Supplementary Data |
| **Academic Editor:** Clearer separation between Results and Discussion required. | Results and Discussion have been fully separated. Observations and descriptive data are confined to the Results section, while interpretation and synthesis are restricted to the Discussion. | Results; Discussion |
| **Academic Editor:** Stronger articulation of implications for heritage management. | The Discussion and Conclusion now explicitly address implications for heritage management under warming permafrost conditions, including the limitations of in situ preservation and the value of legacy excavation data for monitoring degradation. | Discussion; Conclusion |
| **Academic Editor:** Figures require improvements (scale bars, resolution, legends, terminology). | Scale bars have been added where possible; figure resolution improved; Norwegian labels replaced with English; figure legends clarified; panel labels enlarged. The term “diachronic” has been removed to avoid temporal ambiguity. | Figures 1–10 and captions |
| **Reviewer 1:** Clarify acronym NIKU at first mention. | The acronym is now spelled out at first occurrence in the main text. | Introduction |
| **Reviewer 1:** Clarify age-at-death estimation methods. | The Methods section now explicitly states that pubic symphysis morphology was given precedence where observable, with other indicators used complementarily. | Methods: Osteological methods |
| **Reviewer 1:** Typographical correction (“Taphonomic scoring consistently applied…”). | The original text has been removed and replaced with a fully revised section | Methods |
| **Reviewer 1:** Clarify stature phrasing using “range”. | The sentence has been rephrased to avoid ambiguity, using explicit mean values. | Results: Stature |
| **Reviewer 1:** Missing word (“While these supports…”). | Corrected to “While these findings support…”. | Results / Discussion |
| **Reviewer 1:** Repetition of scurvy symptom descriptions. | This section has been substantially rewritten to improve clarity, analytical depth, and consistency with the revised methodological framework. | Results / Discussion |
| **Reviewer 1:** Expand and reference catch-up growth hypothesis. | This section has been expanded, framed more cautiously, and grounded in relevant bioarchaeological and historical literature on adolescent labour, mobility, and growth. | Discussion: Developmental stress |
| **Reviewer 2:** Manuscript lacks analytical depth and reads as a site report. | The manuscript has been reframed as an analytical study. Semi-quantitative data are now presented in tables and appendices, and Results and Discussion are clearly separated. | Methods; Results; Discussion |
| **Reviewer 2:** Use of the term “diachronic” is misleading. | The term has been removed and replaced with explicit reference to comparisons between excavation phases. | Abstract; Introduction; Discussion |
| **Reviewer 2:** Skewed sample size between excavation phases. | The revised manuscript clarifies that the material derives from three excavation campaigns and that the value of the dataset lies in well-documented preservation differences across time and microenvironmental settings, rather than balanced sample sizes. | Methods; Results (RQ1) |
| **Reviewer 2:** Extremely high scurvy prevalence without clear criteria or scoring. | Diagnostic criteria are now explicitly stated with reference to established frameworks. Lesion presence, distribution, and manifestation are described systematically, with individual-level data provided in the Supplementary Data. | Methods; Results (Metabolic disease); Supplementary Data |
| **Reviewer 2:** Enamel hypoplasia observations underdeveloped. | Descriptions of defect types, affected teeth, and frequencies have been expanded and explicitly linked to life-course interpretation and stature data. | Results; Discussion |
| **Reviewer 2:** Isotopic and aDNA data referred to but not integrated. | The manuscript now clearly states that isotopic and aDNA data derive from the same site context, sampled during excavation, and are used here for contextual interpretation. These datasets will be presented in dedicated publications, and their future analytical potential is discussed. | Methods: Biomolecular analyses; Discussion |
| **Reviewer 2:** Clarify whether all skeletal remains were re-analysed. | It is now explicitly stated that all individuals included in the study, including those excavated in the 1980s, were reassessed by the same analyst using the same protocols. | Methods: Osteological methods |
| **Reviewer 2:** Use of “significant” without statistical testing. | All such terminology has been revised (e.g. replaced with “notable”) to avoid implying statistical inference. | Results; Discussion |
| **Journal requirements:** Specimen numbers, repository information, permits, and data availability. | Specimen numbers, repository information, permit details, and an updated Data Availability Statement are now provided. | Methods; Data Availability Statement |
