# Supplementary material for: Skeletons in the permafrost: Exploring climate-driven heritage loss and occupational health at the early modern whaling burial site of Likneset, Svalbard
Source: PLoS One. 2026 May 20;21(5):e0347033. doi: 10.1371/journal.pone.0347033 (PMC13189349; doi:10.1371/journal.pone.0347033)
Supplement: Attachment — Submitted filename: Respons to Reviewers - PONE-D-25-37512R1.docx [file pone.0347033.s006.docx]

**Response to Reviewers**

**Manuscript**: *Skeletons in the Permafrost: Exploring Climate-Driven Heritage Loss and Occupational Health at the Early Modern Whaling Burial Site of Likneset, Svalbard*

**Manuscript ID**: PONE-D-25-37512R1

**Response to Reviewer #2**

**Reviewer comment:**

The reviewer noted a potential inconsistency in the discussion of catch-up growth, suggesting that the interpretation that entry into maritime labour may have improved living conditions appears inconsistent with the broader evidence presented in the manuscript showing substantial physiological stress among Arctic whalers. The reviewer suggested considering alternative explanations such as selective recruitment and survivorship bias.

**Response:**

We thank the reviewer for this thoughtful and helpful comment. The discussion has been revised to clarify that any potential growth recovery would most likely have occurred prior to participation in Arctic whaling, which typically began in late adolescence or early adulthood. The previous formulation suggesting that entry into maritime labour itself may have improved living conditions has therefore been removed.

The revised text now situates this interpretation within a broader life-course perspective, noting that some individuals may have engaged in coastal or regional maritime activities, or other forms of labour, before entering the more physically demanding and environmentally harsh conditions associated with Arctic whaling.

In addition, the discussion now explicitly addresses the potential role of selective recruitment into physically demanding maritime labour and the influence of survivorship bias in shaping the burial assemblage. These revisions clarify that catch-up growth represents only one possible explanation and that alternative interpretations must also be considered in light of the limitations of the available data (see revised section *“Developmental Stress and Life-Course Trajectories”*).
